# Evaluation of the validity of pancreatoduodenectomy in older patients with distal cholangiocarcinoma in terms of recurrence

**DOI:** 10.1007/s00423-025-03694-9

**Published:** 2025-04-03

**Authors:** Wataru Izumo, Hiromichi Kawaida, Ryo Saito, Yuki Nakata, Hidetake Amemiya, Yudai Higuchi, Takashi Nakayama, Kazunori Takahashi, Suguru Maruyama, Koichi Takiguchi, Katsutoshi Shoda, Kensuke Shiraishi, Shinji Furuya, Yoshihiko Kawaguchi, Daisuke Ichikawa

**Affiliations:** Department of Digestive Surgery, University of Yamanashi Hospital, 1110 Shimokato, Chuo-shi, Yamanashi, 409–3898 Japan

**Keywords:** Elderly, Pancreatoduodenectomy, Distal cholangiocarcinoma, Prognosis, Recurrence

## Abstract

**Background:**

This retrospective study investigated the validity of pancreatoduodenectomy (PD) with regard to recurrence in older patients with distal cholangiocarcinoma (DC).

**Methods:**

We compared 28 patients aged ≥ 75 years and 65 patients aged < 75 years who underwent PD for DC, and evaluated the relationship between age, clinicopathological factors, and outcomes.

**Results:**

Postoperative mortality and morbidity rates did not differ between the groups. Although there were no significant differences in 5-year recurrence, disease-specific survival, and overall survival rates between the groups (45.4, 58.1, and 51.7% in patients ≥ 75 years and 50.3%, 62.7%, and 58.1% in patients < 75 years; *P* = 0.73, 0.44, and 0.24, respectively), the median time from recurrence to death (RTD) in older patients was significantly shorter than that in younger patients (0.5 years vs. 1.3 years, *P* = 0.013). In multivariate analysis, age ≥ 75 years (hazard ratio [HR]: 3.0), controlling nutritional status (CONUT) score ≥ 4 (HR: 2.5), poorly-differentiated adenocarcinoma or adenosquamous carcinoma (HR: 3.2), and failure to implement treatment after recurrence (HR: 5.3) were independent risk factors for a short time from RTD. Furthermore, at the time of recurrence, older patients had significantly poorer serum albumin levels, prognostic nutrition index, Glasgow prognostic score, and CONUT score. Age ≥ 75 years (odds ratio: 0.19) was an independent risk factor for implementation of treatment after recurrence.

**Conclusions:**

PD in older patients may be acceptable; however, the median time from RTD was shorter owing to lower nutritional status and rates of treatment implementation after recurrence.

**Supplementary Information:**

The online version contains supplementary material available at 10.1007/s00423-025-03694-9.

## Introduction

Although the incidence of biliary tract carcinoma (BTC) is higher in parts of Asia and South America, its incidence has been increasing in Europe and the United States [1–3]. Based on the anatomical site of origin, BTC is classified into intrahepatic cholangiocarcinoma, perihilar cholangiocarcinoma, gallbladder carcinoma, distal cholangiocarcinoma (DC), and ampullary carcinoma, and each type is thought to have different biological characteristics and malignancy grades [3]. Radical surgical resection is the only curative treatment option for patients with BTC regardless of the anatomical site of origin and pancreatoduodenectomy (PD) with regional lymph node dissection (LND) is the standard operative procedure for DC [4–6]. In recent years, advances in surgical techniques and pre- and postoperative management have improved the safety of PD [7, 8], and the number of patients undergoing PD has been rising in Japan [8]. However, PD remains one of the operative procedures with the highest risk of complications and surgery-related mortality rates among all gastrointestinal surgeries; [8] therefore, the indications for PD should be carefully considered.

Recent advances in medical technology have extended the life expectancy worldwide [9], and the number of older people requiring medical interventions is increasing. The decision regarding whether to provide aggressive medical treatment to older people is complex and involves a variety of factors, including social, economic, and ethical considerations. Furthermore, there is no universal definition of older people or the “elderly.” Japan is one of the countries with the longest life expectancy [10]. People aged 75 years or older are considered “Late-stage elderly” and are covered by a medical insurance system which is different from that covering people aged under 75 years [11]. However, this is merely a systemic categorization and does not convey the essential meaning of “elderly.”

However, surgery in older patients is associated with certain risks because of decrease in tissue fragility, muscle strength, cardiopulmonary function, and physiological reserve capacity with age [12, 13]. Therefore, while it is considered unacceptable to withhold treatment solely because of age, it is an important factor influencing treatment selection. In addition, although immune inflammatory markers and nutritional indices have been reported to affect the prognosis of patients with various gastroenterological diseases [14–16], their role as prognostic predictors in older people remains unclear.

Some studies have reported that the safety and effectiveness of PD in older patients are equivalent to those in younger patients [17–20], while other studies have claimed that they are inferior [21–24], and no definite conclusion has been reached yet. In particular, PD is used for the surgical treatment of carcinoma of the pancreatic head as well as DC. Nevertheless, the prevalence of BTC in patients aged 75 years or older is reportedly higher than that of pancreatic cancer (67.9% vs. 55.4%).^25^ The oncological significance of PD in older patients with DC remains unclear. Furthermore, postoperative treatment and treatment after recurrence are predicted to be difficult in older patients with DC. However, to our knowledge, no studies have compared these factors between older and younger patients with DC. This study aimed to clarify the surgical and oncological validity of PD in older patients with DC, with a focus on recurrence.

## Methods

### Study design

This study was approved by the institutional review board of Yamanashi University (approval number: H30906). The requirement for informed consent was waived owing to the retrospective nature of the study.

Between 2000 and 2021, 132 patients underwent surgical resection of DC at the Department of Digestive Surgery, University of Yamanashi Hospital. We excluded patients who underwent hepatopancreatoduodenectomy (HPD), extrahepatic bile duct resection and reconstruction, residual tumor (R2) resection, and those with an unknown examination status. Finally, 93 patients who underwent curative PD for DC were analyzed retrospectively. In addition, during this study period, the total number of PDs at our institute was 442 cases.

We evaluated the following clinicopathological parameters and their association with recurrence, time from recurrence to death (RTD), and outcomes. Preoperative parameters included age, sex, body mass index, serum levels of total protein, albumin, total cholesterol, triglyceride, C-reactive protein, hemoglobin A1c, neutrophil-to lymphocyte ratio, platelet-to-lymphocyte ratio, lymphocyte-to-monocyte ratio (LMR), prognostic nutrition index (PNI), C-reactive protein/albumin ratio, Glasgow prognostic score (GPS), controlling nutritional status (CONUT) score (definitions of these indicators are shown in Supplemental Table 1), carcinoembryonic antigen, carbohydrate antigen 19 − 9 (CA19-9), and American society of anesthesiologists-physical status [26]. Intraoperative parameters comprised vascular resection, number of LND, duration of surgery, and amount of blood loss, while postoperative parameters included pathological type, local invasion, vascular invasion, lymph node metastasis (LNM), existence of residual tumor, postoperative complication grade, postoperative pancreatic fistula (POPF), delayed gastric emptying (DGE), post- pancreatectomy hemorrhage (PPH), completion of adjuvant chemotherapy, length of postoperative hospital stay, hospital mortality, recurrence, implementation of treatment after recurrence, cause of death, and prognosis. Multivariate analyses of recurrence-free survival (RFS), RTD, disease-specific survival (DSS), and overall survival (OS) were performed by incorporating the above factors.

### Surgical procedure

PD was performed if the patient expressed a wish to undergo PD and if it was medically indicated, after considering the patient’s comorbid status. In patients who underwent PD, the LND region included the area in the hepatoduodenal ligament, area around the common hepatic artery, celiac artery, and superior mesenteric artery, as well as the anterior and posterior surface of the pancreatic head. Portal vein and superior mesenteric vein resections were performed in patients with confirmed or suspected invasion or infiltration of these vessels. The decision regarding the extent of LND and vascular resection was ultimately made at the discretion of the surgeon, while taking into account the patient’s condition and age.

### Definitions

Preoperative laboratory and imaging data were acquired within 1 month of surgery, while laboratory data were measured after biliary decompression with the total bilirubin level < 3.0 mg/dL. The final stage of DC was pathologically defined using the eighth edition of the Union for International Cancer Control criteria [27]. We also included positive surgical margin cases of carcinoma in situ as R1 resections. Postoperative complications were rated according to the Clavien-Dindo classification [28], while POPF, DGE, and PPH were defined according to the International Study Group of Pancreatic Surgery [29–31]. The cut-off values for the above factors were determined using receiver operating characteristic (ROC) analysis and designated as the point at which the area under the ROC curve was the largest for predicting OS. Furthermore, we thought that these cut-off values had high accuracy in predicting OS. Specifically, the time of death was defined as the specific time point used for survival evaluation, and the cut-off value of ROC curve was determined using the Youden Index.

### Adjuvant chemotherapy

Adjuvant chemotherapy was administered for 6 months as a standard practice in patients with stage ≥ I whose general post-surgical condition was stable. Ultimately, the decision to give adjuvant chemotherapy was based on the physician’s and patient’s choice. Adjuvant chemotherapy was previously performed with a variety of regimens at our institute. However, in this study, adjuvant chemotherapy was defined as treatment with capecitabine or S-1 after 2019, when evidence was established [32, 33]. In this study, adjuvant chemotherapy was defined to include only patients who were initiated within 10 weeks after surgery and received more than 80% of the planned dose.

### Follow-up

All patients underwent laboratory examinations, including for tumor markers every 1–3 months for the first 3 years and every 3–6 months after 3–5 years postoperatively, whereas imaging studies were performed every 3 months for the first 3 years and every 3–6 months for 3–5 years postoperatively. The RFS time was calculated from the day of surgery until the diagnosis of recurrence or death of other disease or the last follow-up day if there was no recurrence, whereas RTD was calculated from the time of recurrence until death or the last follow-up date. OS was calculated from the time of surgery until death or the last follow-up date.

### Recurrence

Recurrence was defined as the presence of a tumor on postoperative imaging studies. When two or more sites of recurrence were observed simultaneously, all of the sites were counted. An increase in the tumor marker levels only was not defined as a recurrence in this study. Local recurrence was defined as locoregional recurrence, including that in the nerve plexus. Lymph node recurrence was defined as an increase in the number of nodes on imaging studies. Liver or lung recurrence was defined as a liver or lung mass detected on imaging studies. Peritoneal dissemination recurrence was defined as the detection of an abdominal mass with ascites or peritoneal thickening.

### Treatment after recurrence

The first treatment administered after recurrence was designated as the treatment after recurrence. In this study, only surgical resection, radiation, or drug therapy were included in treatment after recurrence. Drug therapy included only gemcitabine alone, S-1 alone, gemcitabine plus S-1, gemcitabine plus cisplatin (GC), GC plus S-1 (GCS), or GC plus durvalumab (GCD) [34, 35]. Treatment administered to alleviate symptoms alone was not included in treatment after recurrence. Implementation of treatment after recurrence was defined as at least one treatment after recurrence. Ultimately, the decision to undergo treatment after recurrence was based on the physician’s and patient’s choice.

### Statistical analysis

Univariate and multivariate analyses were performed to determine the independent predictors of RTD, DSS, and OS. Survival analyses were performed using the Kaplan-Meier method, log-rank test, and Cox proportional hazards model. Univariate and multivariate logistic regression analyses were performed to determine independent risk factors for implementation of treatment after recurrence in patients with disease recurrence. The Chi-square test was used to analyze the frequency between the two groups. Wilcoxon rank sum test was used to compare continuous variables between the two groups. Only the factors that were significant in the univariate analysis were subjected to multivariate analysis. Statistical significance was set at *P* < 0.05. All analyses were performed using JMP 17.0.0 for Windows (SAS Institute Inc., Cary, NC, USA).

## Results

The preoperative patient characteristics are shown in Table 1. In this study, 30% of the patients were aged ≥ 75 years and 70% were aged < 75 years. There were no significant differences in the pre-, intra-, and postoperative factors other than age, including the levels of various immune-inflammatory markers, nutritional index, frequency of postoperative complications, mortality rates, duration of postoperative hospital stay, and completion of adjuvant chemotherapy between the two groups.

**Table 1:** Preoperative clinicopathological characteristics of the resected patients with distal cholangiocarcinoma

		Age		*P* value
		≥ 75 (*n* = 28)	< 75 (*n* = 65)	
Age (years, range)		78 (75–86)	68 (31–74)	< 0.001
Sex	Male	19 (68%)	49 (75%)	0.46
ECOG-PS	0–1 / ≥ 2	27 (96%) / 1 (4%)	60 (92%) / 5 (8%)	0.43
Preoperative BMI (kg/m^2^, range)		21.6 (18.9–29.3)	21.4 (16.4–29.8)	0.85
Preoperative TP (g/dL, range)		6.9 (5.5–8.4)	6.9 (5.4–8.5)	0.66
Preoperative Alb (g/dL, range)		3.9 (2.1–4.6)	3.8 (2.7–4.9)	0.64
Preoperative TC (mg/dL, range)		162 (93–256)	171 (80–378)	0.17
Preoperative TG (mg/dL, range)		104 (51–178)	111 (46–305)	0.73
Preoperative CRP (mg/dL, range)		0.24 (0.1–2.9)	0.21 (0.1–13.4)	0.20
Preoperative HbA1c (%, range)		5.6 (4.5–7.9)	5.7 (4.4–12.1)	0.22
Preoperative NLR (range)		2.2 (1.1–21.6)	2.4 (0.78–11.7)	0.61
Preoperative PLR (range)		151.3 (64.5-1014.4)	170.2 (66.8-511.1)	0.80
Preoperative LMR (range)		4.3 (0.89–7.7)	4.0 (1.3–10.4)	0.16
Preoperative PNI (range)		44.6 (22.1–56.2)	44.9 (33.1–64.7)	0.59
Preoperative CAR (range)		0.061 (0.022-1.4)	0.057 (0.020–3.9)	0.31
Preoperative GPS score	0 / 1 / 2	19 (68%) / 5 (18%) / 4 (14%)	40 (61%) / 16 (25%) / 9 (14%)	0.76
Preoperative CONUT score	< 4 / ≥ 4	19 (68%) / 9 (32%)	43 (66%) / 22 (34%)	0.87
Preoperative CEA (ng/mL, range)		2.2 (0.8–18.5)	2.2 (0.8–45.9)	0.81
Preoperative CA19-9 (U/mL, range)		27.8 (0.6–207)	31.8 (0.6-44000)	0.30
ASA-PS	Class 1 / 2 / 3	1 (4%) / 19 (57%) / 11 (39%)	2 (3%) / 47 (72%) / 16 (25%)	0.35
Vascular resection	With	2 (7%)	3 (5%)	0.63
Number of dissected lymph node (range)		7 (2–42)	11 (0–32)	0.25
Operative time (min, range)		434 (256–720)	473 (293–703)	0.21
Blood loss (ml, range)		532 (133–2312)	652 (247–2831)	0.84
Pathological type	wel / mod / por / ase	8 (29%) / 13 (46%) / 5 (18%) / 2 (7%)	32 (49%) / 21 (32%) / 11 (17%) 1 (2%)	0.18
Stage	I; / IIA / IIIB / IIIA / IIIB	4 (14%) / 13 (46%) / 8 (29%) / 3 (11%) / 0 (0%)	5 (8%) / 25 (38%) / 27 (41%) / 7 (11%) / 1 (2%)	0.60
Residual tumor	R0	24 (86%)	44 (68%)	0.061
Postoperative complication grade	≥ III	10 (36%)	19 (30%)	0.57
POPF	B or C	9 (32%)	16 (25%)	0.46
DGE	B or C	3 (11%)	7 (11%)	0.99
PPH	B or C	1 (4%)	0 (0%)	0.12
Adjuvant chemotherapy	With	3 (11%)	10 (15%)	0.54
Postoperative hospital stay (day, range)		28 (9–71)	27 (9–76)	0.79
Recurrence		14 (50%)	34 (52%)	0.84
Liver		7 (50%)	11 (32%)	
Lymph node		5 (36%)	9 (26%)	
Local recurrence		3 (21%)	8 (24%)	
Lung		0 (0%)	7 (21%)	
Dissemination		2 (14%)	3 (9%)	
Dead from distal cholangiocarcinoma		11 (39%)	24 (37%)	0.83
Dead from another disease		5 (18%)	8 (12%)	0.49
Postoperative observation period (year, range)		2.4 (0.2–14.7)	3.6 (0.28-20.0)	0.12
In hospital mortality		0 (0%)	0 (0%)	N.A

Abbreviations: ECOG PS: Eastern Cooperative Oncology Group Performance Status, BMI: body mass index, TP: total protein, Alb: albumin, TC: total cholesterol, TG: triglyceride, CRP: C-reactive protein, HbA1c: hemoglobin A1c, NLR: neutrophil-lymphocyte ratio, PLR: platelet-lymphocyte ratio, LMR: Lymphocyte/Monocyte ratio, PNI: prognostic nutrition index, CAR: C-reactive protein-albumin ratio, GPS: glasgow prognostic score, CONUT: controlling nutritional status, CEA: carcinoembryonic antigen, CA19-9: carbohydrate antigen 19 − 9, ASA-PS: American society of anesthesiologists-physical status, PD: pancreaticoduodenectomy, EHBD: extrahepatic bile duct resection, wel: well differentiated adenocarcinoma, mod: moderately differentiated adenocarcinoma, por: poorly differentiated adenocarcinoma, asc: adenosquamous carcinoma, POPF: postoperative pancreatic fistula grade, DGE: delayed gastric empty grade, PPH: post-pancreatectomy hemorrhage, N.A: not available

### Recurrence rate, RFS, DSS, and OS

**Fig. 1:**
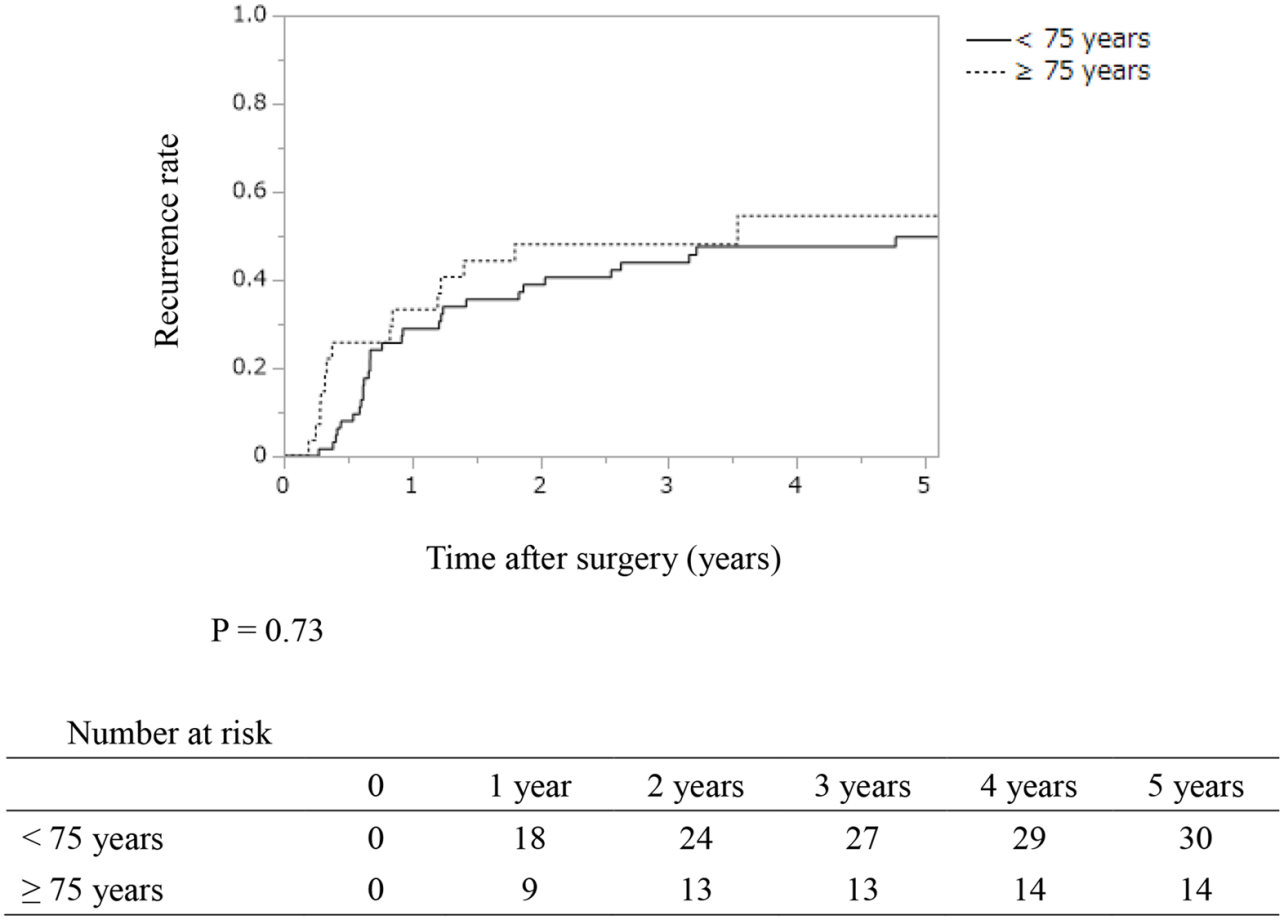
Kaplan–Meier analyses of recurrence rates in resected patients with distal cholangiocarcinoma according to their ages. The 1-, 3-, and 5-year recurrence rates were 29.0%, 43.9%, and 49.7% in patients aged < 75 years, and 33.2%, 48.1%, and 54.6% in those aged ≥ 75 years, respectively. There were no significant differences between the two groups (*P* = 0.73)

**Fig. 2:**
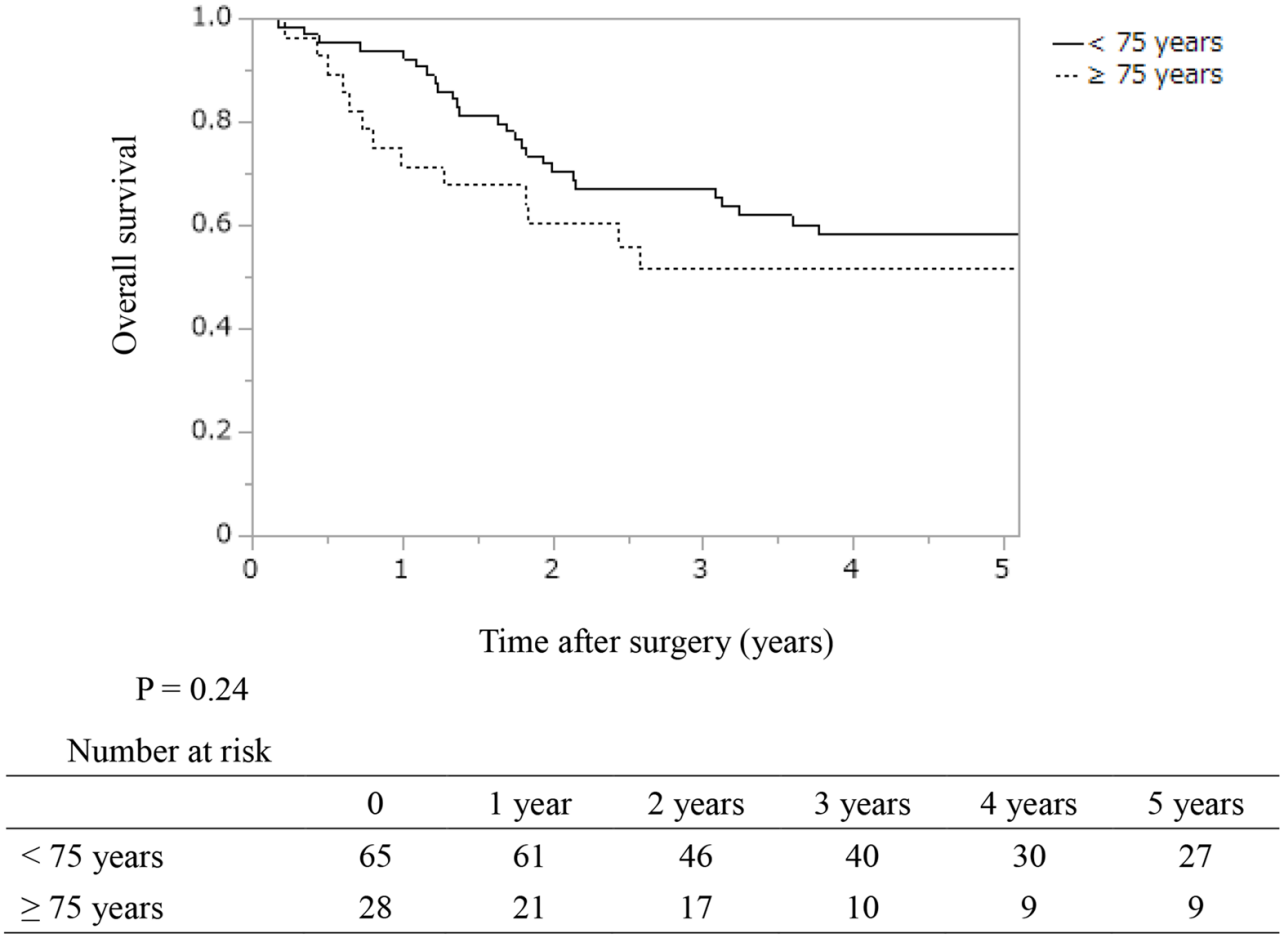
Kaplan–Meier analyses of overall survival in patients who underwent resection of distal cholangiocarcinoma according to their ages. The 1-, 3-, and 5-year overall survival rates were 93.8%, 67.2%, and 58.1% in patients aged < 75 years, and 71.4%, 51.7%, and 51.7% in those aged ≥ 75 years, respectively. There were no significant differences between the two groups (*P* = 0.24)

The recurrence rate was 52% (48/93) in all patients: 50% (14/28) in patients aged ≥ 75 years and 52% (34/65) in patients aged < 75 years, with no significant difference between the two groups (*P* = 0.84). The recurrence sites (with duplication) were mainly the liver (18 patients, 35%), followed by the lymph nodes (14 patients, 29%), local recurrence (11 patients, 23%), lungs (seven patients, 15%), and dissemination (five patients, 10%). There were no significant differences in the 5-year recurrence rate and median recurrent time between the two groups (54.6% and 3.5 years in patients ≥ 75 years; 49.7% and 5.1 years in patients < 75 years; *P* = 0.73) (Fig. 1). There were no significant differences in the 5-year RFS, DSS, and OS between the two groups (45.4, 58.1, and 51.7% in patients ≥ 75 years; 50.3%, 62.7%, and 58.1% in patients < 75 years; *P* = 0.73, 0.44, and 0.24, respectively) (Fig. 2).

### Poor prognostic factors in resected patients

In multivariate analyses, a preoperative CONUT score ≥ 4, poorly-differentiated adenocarcinoma or adenosquamous carcinoma, existence of LNM, and residual tumor (R1 resection) were poor prognostic factors for OS (hazard ratio [HR] (95% confidence interval [95% CI]): 2.9 (1.3–6.7), 2.6 (1.2–5.5), 2.2 (1.1–5.4), and 2.2 (1.2–4.2), respectively), while age ≥ 75 years was not a significant adverse prognostic factor (Table 2). Similarly, a preoperative CONUT score ≥ 4, poorly-differentiated adenocarcinoma or adenosquamous carcinoma, and existence of LNM were poor prognostic factors for DSS (HR (95% CI): 2.9 (1.3–6.2), 2.7 (1.2–6.2), and 2.5 (1.1–5.6), respectively), while age ≥ 75 years was not a significant adverse prognostic factor (Supplemental Table 2).

**Table 2:** Univariate and multivariate analyses of prognostic factors for overall survival in resected patients with distal cholangiocarcinoma

			Univariate			Multivariate	
Prognostic factors	Definition	n	OS		P value	Hazard ratio (95% CI)	P value
			3-years	5-years			
Age (years)	< 75	65	67.2	58.1	0.24		
	≥ 75	28	51.7	51.7			
Preoperative PNI	≥ 40.5	71	71.4	66.0	< 0.001	1.0	0.47
	< 40.5	22	34.3	22.8		1.8 (0.66–5.1)	
Preoperative CAR	< 0.054	44	74.6	64.7	0.059		
	≥ 0.054	49	52.1	47.5			
Preoperative GPS score	0–1	80	66.7	58.5	0.023	1.0	0.068
	2	13	38.5	38.5		1.9 (0.88–5.2)	
Preoperative CONUT score	0–3	62	73.7	69.5	< 0.001	1.0	0.010
	≥ 4	31	40.8	29.2		2.9 (1.3–6.7)	
Preoperative CEA (ng/mL)	< 10.5	89	63.3	56.1	0.77		
	≥ 10.5	4	50.0	50.0			
Preoperative CA19-9 (U/mL)	< 95.7	73	66.3	59.2	0.051		
	≥ 95.7	20	50.0	44.4			
ASA-PS	Class 1–2	66	68.9	63.2	0.015	1.0	0.58
	Class 3	27	48.2	37.9		1.2 (0.61–2.4)	
Pathological type	wel + mod	74	72.4	65.4	< 0.001	1.0	0.016
	por + asc	19	26.3	21.1		2.6 (1.2–5.5)	
T factor	T 1–2	66	70.4	62.2	0.0090	1.0	0.20
	T 3–4	27	44.4	40.4		1.7 (0.76–3.6)	
Lymph node metastasis	Negative	53	70.5	62.9	0.011	1.0	0.036
	Positive	40	52.5	46.6		2.2 (1.1–5.4)	
Residual tumor	R0	78	68.4	62.7	0.0038	1.0	0.012
	R1	25	48.0	40.0		2.2 (1.2–4.2)	
Adjuvant chemotherapy	With	13	61.6	46.2	0.89		
	Without	80	62.9	56.9			

Abbreviations: OS: overall survival rate, CI: confidence interval, PNI: prognostic nutrition index, CAR: C-reactive protein-albumin ratio, GPS: glasgow prognostic score, CONUT: controlling nutritional status, CEA: carcinoembryonic antigen, CA19-9: carbohydrate antigen 19 − 9, ASA-PS: American society of anesthesiologists-physical status, wel: well differentiated adenocarcinoma, mod: moderately differentiated adenocarcinoma, por: poorly differentiated adenocarcinoma, asc: adenosquamous carcinoma

### Recurrence status and risk factors for short RTD

**Fig. 3:**
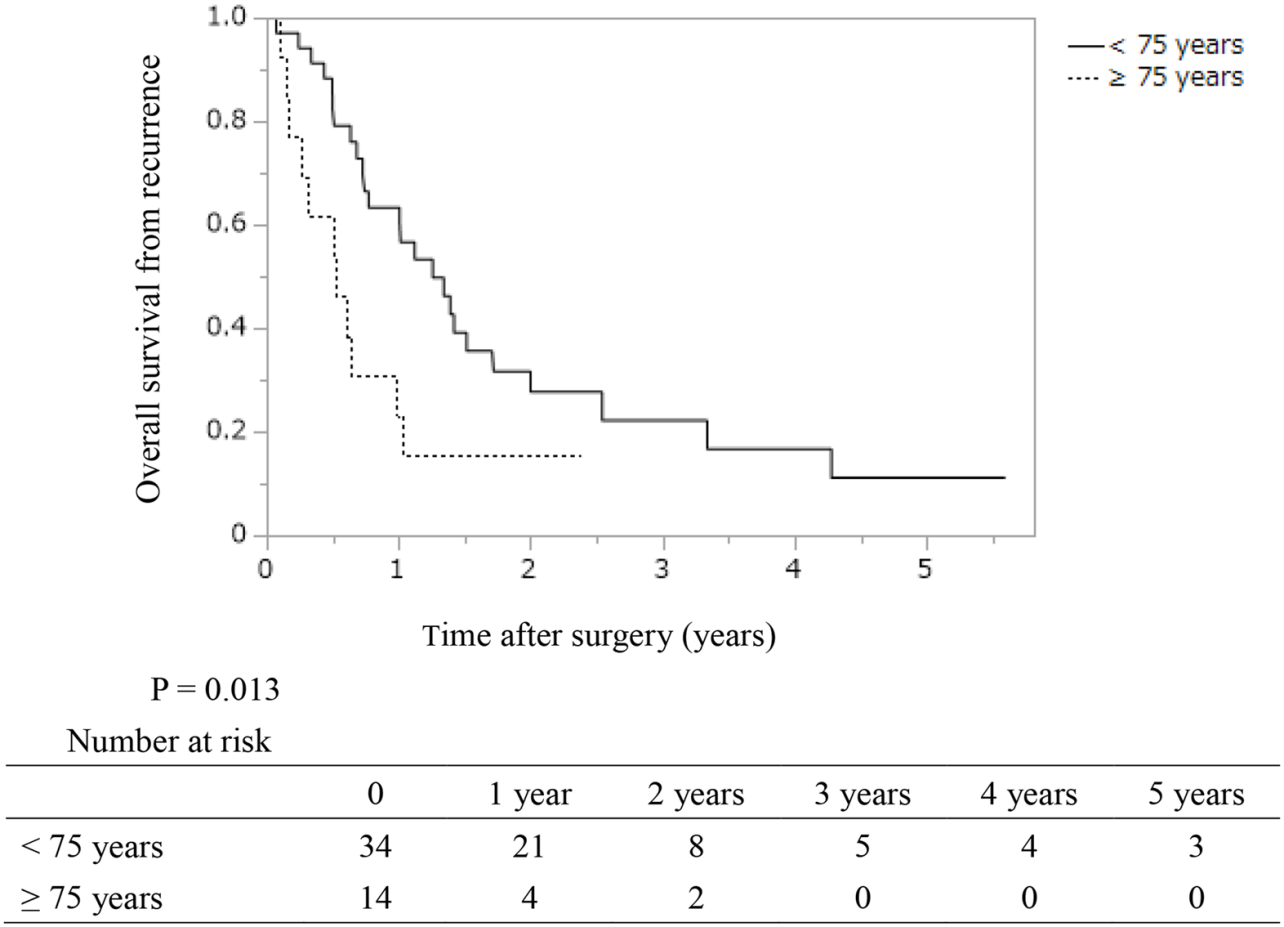
Kaplan–Meier analyses of survival time from recurrence to death in resected patients with distal extrahepatic bile duct carcinoma according to their ages. The 1- and 3-year survival rates after recurrence were 63.3% and 22.1% (median survival time from recurrence to death: 1.3 years) in patients aged < 75 years and 23.1% and not available (median survival time from recurrence to death: 0.5 years) in those aged ≥ 75 years, respectively. The median time from recurrence to death in patients ≥ 75 years was significantly shorter than that in patients < 75 years (*P* = 0.013)

The patient characteristics at the time of recurrence are shown in Table 3. At the time of recurrence, serum albumin level, LMR, and PNI were significantly lower and the GPS and CONUT score were significantly higher in patients ≥ 75 years (*P* = 0.025, 0.048, 0.014, 0.025, and 0.0075, respectively). Moreover, the treatment rate after recurrence was also significantly lower in older patients (36% in patients ≥ 75 years, 79% in patients < 75 years; *P* = 0.0040). The median time from RTD in patients aged ≥ 75 years was significantly shorter than that in patients aged < 75 years (0.5 years vs. 1.3 years, *P* = 0.013) (Fig. 3). In multivariate analysis, age ≥ 75 years, CONUT score ≥ 4, poorly-differentiated adenocarcinoma or adenosquamous carcinoma, and failure to implement treatment after recurrence were independent risk factors for a short time from RTD (HR (95% CI): 3.0 (1.2–6.6), 2.5 (1.1–5.6), 3.2 (1.4–7.3), and 5.3 (2.0–14.0), respectively) (Table 4). When assessing outcomes using only age ≥ 75 years and CONUT score ≥ 4, which can be easily assessed preoperatively, recurrent patients with risk scores of 1–2 had a significantly shorter time from RTD than patients with a risk score of zero (median RTD: 0.5 and 1.4 years, *P* = 0.0010) (Supplemental Fig. 1). Furthermore, in multivariate analyses, only age ≥ 75 years (odds ratio (95% CI): 0.19 (0.042–0.84), *P* = 0.028) was an independent risk factor for implementation of treatment after recurrence (Table 5).

**Table 3:** Clinicopathological characteristics of the recurrent patients at the time of recurrence

		Age		*P* value
		≥ 75 (*n*=14)	< 75 (*n*=34)	
Age at the time of first surgery (years, range)		79 (75-86)	69 (31-74)	< 0.001
Sex	Male	7 (50%)	28 (82%)	0.026
ECOG PS	0-1 / ≥ 2	9 (64%) / 5 (36%)	30 (88%) / 4 (12%)	0.063
Age-adjusted Charlson comorbidity index	≤ 10 / ≥ 11	8 (57%) / 6 (43%)	26 (76%) / 8 (24%)	0.19
TP at the time of recurrence (g/dL, range)		6.7 (6.0-7.1)	6.8 (5.8-7.7)	0.45
Alb at the time of recurrence (g/dL, range)		3.4 (2.4-4.1)	3.9 (2.1-4.7)	0.025
TC at the time of recurrence (mg/dL, range)		140 (66-227)	147 (99-266)	0.52
TG at the time of recurrence (mg/dL, range)		94 (45-158)	94 (41-277)	0.67
CRP at the time of recurrence (mg/dL, range)		0.56 (0.1-9.8)	0.13 (0.1-18.9)	0.96
NLR at the time of recurrence (range)		2.9 (0.76-8.9)	2.2 (0.68-17.8)	0.43
PLR at the time of recurrence (range)		176.6 (71.3-368.8)	165.5 (22.4-377.5)	0.19
LMR at the time of recurrence (range)		2.7 (0.82-5.8)	4.0 (1.0-6.8)	0.048
PNI at the time of recurrence (range)		38.0 (28.4-49.1)	45.4 (29.6-53.1)	0.014
CAR at the time of recurrence (range)		0.18 (0.024-4.1)	0.035 (0.023-7.8)	0.96
GPS score at the time of recurrence	0-1 / 2	5 (36%) / 9 (64%)	24 (71%) / 10 (29%)	0.025
CONUT score at the time of recurrence	0-4 / ≥ 5	6 (43%) / 8 (57%)	28 (82%) / 6 (18%)	0.0075
CEA at the time of recurrence (ng/mL, range)		4.0 (1.4-41.9)	3.9 (0.7-17.4)	0.13
CA19-9 at the time of recurrence (U/mL, range)		82.6 (5.1-1856)	51.7 (5.5-1162)	0.39
Pathological type	wel or mod / por or asc	9 (64%) / 5 (36%)	25 (74%) / 9 (27%)	0.53
Treatment after recurrence	With	5 (36%)	27 (79%)	0.0040
	Chemotherapy	5 (100%)	25 (92%)	
	Radiation	0 (0%)	1 (4%)	
	Surgical resection	0 (0%)	1 (4%)	
Dead from distal cholangiocarcinoma		11 (79%)	23 (68%)	0.44
Dead from another disease		0 (0%)	2 (6%)	0.23

Abbreviations: ECOG PS: Eastern Cooperative Oncology Group Performance Status, TP: total protein, Alb: albumin, TC: total cholesterol, TG: triglyceride, CRP: C-reactive protein, NLR: neutrophil-lymphocyte ratio, PLR: platelet-lymphocyte ratio, LMR: Lymphocyte/Monocyte ratio, PNI: prognostic nutrition index, CAR: C-reactive protein-albumin ratio, GPS: glasgow prognostic score, CONUT: controlling nutritional status, CEA: carcinoembryonic antigen, CA19-9: carbohydrate antigen 19 − 9, wel: well differentiated adenocarcinoma, mod: moderately differentiated adenocarcinoma, por: poorly differentiated adenocarcinoma, asc: adenosquamous carcinoma

**Table 4:** Univariate and multivariate analyses of risk factors for short time from recurrence to death in recurrent patients with distal cholangiocarcinoma

			Univariate		Multivariate	
Prognostic factors	Definition	n	Median RTD (years) (95% CI)	P value	Hazard ratio (95% CI)	P value
Age at the time of first surgery (years)	< 75	34	1.3 (0.74-1.7)	0.013	1.0	0.032
	≥ 75	14	0.53 (0.17-0.99)		3.0 (1.2-6.6)	
PNI at the time of recurrence	≥ 40.5	30	1.4 (0.73-2.5)	0.0033	1.0	0.95
	< 40.5	18	0.61 (0.17-0.99)		1.1 (0.25-4.4)	
CAR at the time of recurrence	< 0.054	24	1.4 (0.73-3.3)	0.0065	1.0	0.14
	≥ 0.054	24	0.76 (0.50-1.1)		2.2 (0.78-6.0)	
GPS score at the time of recurrence	0-1	41	1.0 (0.68-1.5)	0.026	1.0	0.98
	2	7	0.50 (0.072-1.3)		1.0 (0.31-3.3)	
CONUT score at the time of recurrence	0-3	28	1.4 (0.74-2.5)	0.0025	1.0	0.041
	≥ 4	20	0.51 (0.24-0.77)		2.5 (1.1-5.6)	
CEA at the time of recurrence (ng/mL)	< 10.5	44	1.0 (0.64-1.4)	0.017	1.0	0.67
	≥ 10.5	4	0.5 (0.14-0.73)		1.4 (0.29-6.6)	
CA19-9 at the time of recurrence (U/mL)	< 95.7	30	1.4 (0.64-2.5)	0.015	1.0	0.30
	≥ 95.7	18	0.76 (0.44-1.0)		1.5 (0.68-3.5)	
Pathological type	wel + mod	34	1.3 (0.68-1.7)	0.030	1.0	0.006
	por + asc	14	0.57 (0.24-1.1)		3.2 (1.4-7.3)	
T factor	T 1-2	30	1.1 (0.68-1.5)	0.43		
	T 3-4	18	0.69 (0.26-1.4)			
Lymph node metastasis	Negative	20	1.3 (0.51-1.7)	0.83		
	Positive	28	0.77 (0.53-1.4)			
Residual tumor	R0	32	1.0 (0.64-1.5)	0.17		
	R1	16	0.73 (0.50-1.4)			
Adjuvant chemotherapy	With	9	1.4 (0.44-2.5)	0.26		
	Without	39	0.99 (0.53-1.3)			
Implementation of treatment after recurrence	Implemented	32	1.4 (1.0-2.5)	< 0.001	1.0	< 0.001
	Not implemented	14	0.50 (0.14-0.64)		5.3 (2.0-14.0)	

Abbreviations: RTD: time from recurrence to death, CI: confidence interval, PNI: prognostic nutrition index, CAR: C-reactive protein-albumin ratio, GPS: glasgow prognostic score, CONUT: controlling nutritional status, CEA: carcinoembryonic antigen, CA19-9: carbohydrate antigen 19-9, wel: well differentiated adenocarcinoma, mod: moderately differentiated adenocarcinoma, por: poorly differentiated adenocarcinoma, asc: adenosquamous carcinoma

**Table 5:** Univariate and multivariate analyses of risk factors for implementation of treatment after recurrence in patients with disease recurrence

				Univariate		Multivariate	
Prognostic factors	Definition	n	Implement of treatment	OR (95% CI)	P value	OR (95% CI)	P value
Age at the time of first surgery (years)	< 75	34	27 (79%)	1.0	0.0057	1.0	0.028
	≥ 75	14	5 (36%)	0.14 (0.036-0.57)		0.19 (0.042-0.84)	
PNI at the time of recurrence	≥ 40.5	30	24 (80%)	1.0	0.012	1.0	0.56
	< 40.5	18	8 (44%)	0.2 (0.055-0.73)		0.47 (0.038-5.9)	
CAR at the time of recurrence	< 0.054	24	18 (75%)	1.0	0.22		
	≥ 0.054	24	14 (58%)	0.47 (0.14-1.6)			
GPS score at the time of recurrence	0-1	41	28 (68%)	1.0	0.57		
	2	7	4 (57%)	0.62 (0.12-3.2)			
CONUT score at the time of recurrence	0-3	28	22 (79%)	1.0	0.043	1.0	0.67
	≥ 4	20	10 (50%)	0.27 (0.077-0.96)		0.59 (0.049-7.0)	
CEA at the time of recurrence (ng/mL)	< 10.5	44	32 (73%)	1.0	N.A		
	≥ 10.5	4	0 (0%)	N.A			
CA19-9 at the time of recurrence (U/mL)	< 95.7	30	21 (70%)	1.0	0.53		
	≥ 95.7	18	11 (61%)	0.67 (0.20-2.3)			
Adjuvant chemotherapy	With	9	8 (89%)	1.0	0.15		
	Without	39	24 (62%)	0.2 (0.023-1.8)			

Abbreviations: OR: odds ratio, CI: confidence interval, PNI: prognostic nutrition index, CAR: C-reactive protein-albumin ratio, GPS: glasgow prognostic score, CONUT: controlling nutritional status, CEA: carcinoembryonic antigen, CA19-9: carbohydrate antigen 19 − 9, wel: well differentiated adenocarcinoma, mod: moderately differentiated adenocarcinoma, por: poorly differentiated adenocarcinoma, asc: adenosquamous carcinoma, N.A: not available

## Discussion

This study revealed no significant differences in postoperative mortality and morbidity, RFS, DSS, and OS between patients aged ≥ 75 years and those aged < 75 years who underwent PD. In contrast, at the time of recurrence, immuno-inflammatory indexes and nutritional indicators were significantly worse in patients aged ≥ 75 years and the median survival time from RTD was significantly shorter compared to patients aged < 75 years. In addition, age ≥ 75 years was an independent risk factor for not receiving treatment after recurrence. These findings suggest that older patients with recurrence may have a poor general condition due to various factors as well as a shorter RTD due to difficulty in implementing treatment after recurrence. These findings may be useful when considering treatment strategies for older patients with DC.

BTC is one of the gastrointestinal cancers with a poor prognosis, and the only curative treatment is surgical resection. However, recurrence is often observed even after radical resection [6] and the 5-year survival rate in patients with BTC after curative resection has been reported to be as low as approximately 20%.^36^ In Japan, BTC is the sixth leading cause of cancer-related deaths, accounting for approximately 18 000 deaths per year [37]. The surgical methods for DC, which is a type of BTC, include extrahepatic bile duct resection, PD, and HPD. With regard to the surgery-related complications and prognosis, PD with regional LND is currently the standard surgical procedure for resectable DC [4–6]. PD is also used for the treatment of pancreatic head and ampullary carcinomas. Therefore, it is important to investigate the safety and efficacy of PD in older patients. Despite the recent advances in medical technology, PD carries a certain degree of morbidity and mortality [7, 8, 17–24]. As mentioned earlier, there are conflicting reports regarding the safety and effectiveness of PD in older patients as compared to younger patients. Studies that claimed that PD was as safe in older patients as in younger patients were mainly conducted in high-volume centers that provide high-quality medical care [17–20]. Conversely, a real-world large-scale study in Japan revealed that the overall hospital mortality rate after PD was 2.2%, with patients aged 70 years or older having higher postoperative mortality and complication rates, and longer length of postoperative hospital stay than those aged 70 years or younger [21]. Thus, the safety of PD in older patients remains a crucial unresolved issue in these times of expanding life expectancy, as it varies depending on patient background characteristics and study population. In this study, the hospital mortality rate was favorable, and the length of postoperative hospital stay was shorter than that reported in the aforementioned large-scale study, indicating that the results of this study are safer and more reliable.

In cases of recurrence after radical resection, multidisciplinary treatment is administered, which mainly comprises drug therapy, but the median OS remains low at approximately 1 year [4, 32, 33]. Although recent studies have demonstrated improvement in prognosis of patients undergoing GCD and GCS therapy compared to conventional treatment regimens such as GC therapy, the prognosis remains poor, with a median survival time of approximately 12.8 and 16.2 months with GCD and GCS therapy, respectively [34, 35]. Most of the previous clinical trials excluded and/or included only a small number of older patients; [32–35] therefore, it remains unclear whether the safety and effectiveness of drug therapy is similar between older and younger patients with DC. Previous studies have reported that poor cognitive and physical function and performance status in older patients make it difficult to continue chemotherapy [38, 39]. However, the initiation and continuation of chemotherapy should take into account multiple factors in addition to age, and the decision to initiate chemotherapy should be based on the patient’s general condition using a numerical index. Immune-inflammatory and nutritional indicators have been reported as predictors of postoperative prognosis and chemotherapy efficacy in some cancers [14–16]. Some previous literatures also reported that preoperative GPS score and PNI are independent prognostic factors in patients with resected BTC [14, 40–43]. Furthermore, it has been reported that preoperative NLR and PLR may be useful predictors of prognosis after resection of BTC [44]. These variables can be readily evaluated preoperatively and may be potentially useful for predicting therapeutic treatability after recurrence. To our knowledge, no previous study has examined the efficacy of these indicators in predicting treatment suitability after recurrence in patients with DC. This study examined various indicators and demonstrated that CONUT score is an important patient factor that affects the treatment after recurrence. In recent years, the concept of frailty has been reported as an indicator of the clinical performance status, and a previous study showed that improved prediction of adverse postoperative outcomes in patients with PD by combining frailty with other factors.^45^ Although age was identified as a risk factor for a short survival time from RTD and treatment failure after recurrence in this study, age is merely one factor, and there is a need to explore other patient background characteristics, which are currently difficult to quantify. Future studies should aim to elucidate these factors based on various preoperative measurable parameters that influence treatment after recurrence in older patients with DC to aid clinical decision-making regardless of age. Based on the results of this study, it is possibility that improving the immune-inflammatory and nutritional status may extend RTD in patients with resected BTC. In addition, future clinical research is necessary to verify whether improving these indicators through pre- and postoperative interventions will lead to extended survival.

This study has some limitations. First, over the 24-year study period, patients received different diagnostic tests and treatments, including various chemotherapy regimens and pre-, intra-, and postoperative management plans, owing to advances in these techniques over time. Thus, a selection bias cannot be ruled out, especially for surgical indications and procedures. These variations may have skewed the outcomes of patients treated during different periods of the study. In addition, our study may have included cases with insufficient observation period. Second, the number of cases and recurrence/death events was insufficient compared to the number of items used in the multivariate analysis. Moreover, we included several factors that may be highly correlated in the multivariate analysis and there may be statistical effects. Finally, this was a retrospective study performed at a single institution, which may limit the generalizability of the results. However, this study was conducted as a preliminary pilot study to clarify the validity of surgical resection in older patients with DC. The results of this study are satisfactory in that they lay the groundwork for further research. Large scale observational studies are needed to confirm the findings of this study.

In conclusion, we demonstrated that PD in patients with DC aged ≥ 75 years may be tolerated, but the treatment of recurrent disease in these patients is difficult and they have a shorter RTD.

## Electronic supplementary material

Below is the link to the electronic supplementary material.

Supplementary Material 1

## Data Availability

No datasets were generated or analysed during the current study.
