# Supplementary material for: Evaluation of the validity of pancreatoduodenectomy in older patients with distal cholangiocarcinoma in terms of recurrence
Source: Langenbecks Arch Surg. 2025 Apr 3;410(1):119. doi: 10.1007/s00423-025-03694-9 (PMC11968497; doi:10.1007/s00423-025-03694-9)
Supplement: Supplementary file 1 — Supplementary Material 1 [file 423_2025_3694_MOESM1_ESM.docx]

Supplemental Table 1: Definitions of indicators

| Pre-chemotherapeutic indicators | Abbreviation | Formula |
| --- | --- | --- |
| Neutrophil-Lymphocyte Ratio | NLR | Neutrophil / Lymphocyte |
| Platelet-Lymphocyte Ratio | PLR | Platelet / Lymphocyte |
| Prognostic Nutritional Index | PNI | 10 × Alb + 0.005 × Lymphocyte |

| Controlling Nutritional Status (CONUT) | | | | |
| --- | --- | --- | --- | --- |
| Alb (g / dL) | Alb ≥ 3.5 | 3.0 ≤ Alb < 3.5 | 2.5 ≤ Alb < 3.0 | Alb < 2.5 |
| Alb score | 0 | 2 | 4 | 6 |
| TLC (/ μL) | TLC ≥ 1600 | 1200 ≤ TLC < 1600 | 800 ≤ TLC < 1200 | TLC < 800 |
| TLC score | 0 | 1 | 2 | 3 |
| T-cho (mg / dL) | T-cho ≥ 180 | 140 ≤ T-cho < 180 | 100 ≤ T-cho < 140 | T-cho < 100 |
| T-cho score | 0 | 1 | 2 | 3 |
| CONUT score = Alb score + TLC score + T-cho score | | | | |

| GPS score (GPS) | |
| --- | --- |
| CRP ≤ 1.0 mg/dL and Alb ≥ 3.5 g/dL | 0 |
| CRP > 1.0 mg/dL or Alb < 3.5 g/dL | 1 |
| CRP > 1.0 mg/dL and Alb < 3.5 g/dL | 2 |

Alb: serum albumin level; TLC: total lymphocyte count; T-cho: serum total cholesterol level.

Supplemental Table 2. Univariate and multivariate analyses of prognostic factors for disease-specific survival in resected patients with distal cholangiocarcinoma

|  |  |  | Univariate | | | Multivariate | |
| --- | --- | --- | --- | --- | --- | --- | --- |
| Prognostic factors | Definition | n | DSS | | P value | Hazard ratio  (95% CI) | P value |
|  |  |  | 3-years | 5-years |  |  |  |
| Age (years) at the time of surgery | < 75 | 65 | 70.6 | 62.7 | 0.44 |  |  |
|  | ≥ 75 | 28 | 58.1 | 58.1 |  |  |  |
| Preoperative PNI (range) | ≥ 40.5 | 71 | 74.7 | 70.8 | 0.0011 | 1.0 | 0.32 |
|  | < 40.5 | 22 | 41.0 | 27.4 |  | 1.9 (0.53-7.1) |  |
| Preoperative CAR (range) | < 0.054 | 44 | 76.3 | 69.2 | 0.097 |  |  |
|  | ≥ 0.054 | 49 | 58.0 | 52.9 |  |  |  |
| Preoperative GPS score | 0-1 | 80 | 69.9 | 62.7 | 0.17 |  |  |
|  | 2 | 13 | 46.9 | 46.9 |  |  |  |
| Preoperative CONUT score | 0-3 | 62 | 74.9 | 70.6 | 0.0098 | 1.0 | 0.0082 |
|  | ≥ 4 | 31 | 49.1 | 38.7 |  | 2.9 (1.3-6.2) |  |
| Preoperative CEA (ng/mL) | < 10.5 | 89 | 67.7 | 61.3 | 0.31 |  |  |
|  | ≥ 10.5 | 4 | 50.0 | 50.0 |  |  |  |
| Preoperative CA19-9 (U/mL) | < 95.7 | 73 | 71.9 | 65.9 | 0.014 | 1.0 | 0.086 |
|  | ≥ 95.7 | 20 | 50.0 | 44.4 |  | 2.2 (0.89-5.6) |  |
| ASA-PS | Class 1-2 | 66 | 74.0 | 69.7 | 0.0072 | 1.0 | 0.40 |
|  | Class 3 | 27 | 50.0 | 39.4 |  | 1.4 (0.64-3.1) |  |
| Pathological type | wel + mod | 74 | 75.5 | 69.9 | < 0.001 | 1.0 | 0.021 |
|  | por + asc | 19 | 33.6 | 26.8 |  | 2.7 (1.2-6.2) |  |
| T factor | T 0-2 | 66 | 73.9 | 67.1 | 0.013 | 1.0 | 0.63 |
|  | T 3-4 | 27 | 50.2 | 45.6 |  | 1.2 (0.52-2.9) |  |
| Lymph node metastasis | Negative | 53 | 77.3 | 71.4 | 0.0094 | 1.0 | 0.042 |
|  | Positive | 40 | 53.9 | 47.8 |  | 2.5 (1.1-5.6) |  |
| Residual tumor | R0 | 78 | 70.5 | 66.4 | 0.0035 | 1.0 | 0.80 |
|  | R1 | 25 | 57.1 | 47.6 |  | 1.1 (0.50-2.5) |  |
| Adjuvant chemotherapy | With | 13 | 61.6 | 46.2 | 0.64 |  |  |
|  | Without | 80 | 67.9 | 62.9 |  |  |  |

Abbreviations: DSS: disease-specific survival, PNI: prognostic nutrition index, CAR: C-reactive protein-albumin ratio, GPS: glasgow prognostic score, CONUT: controlling nutritional status, CEA: carcinoembryonic antigen, CA19-9: carbohydrate antigen 19-9, ASA-PS: American society of anesthesiologists-physical status, wel: well differentiated adenocarcinoma, mod: moderately differentiated adenocarcinoma, por: poorly differentiated adenocarcinoma, asc: adenosquamous carcinoma

Supplemental Figure 1. Kaplan–Meier analyses of overall survival time from recurrence to death in resected patients with distal cholangiocarcinoma according to risk score


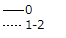

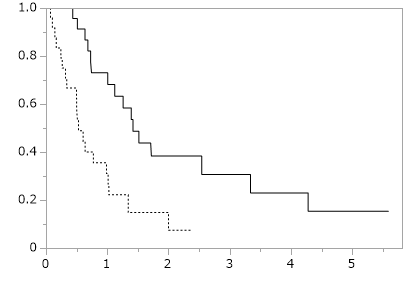


Overall survival from recurrence

Time after recurrence (years)

P = 0.0010

Number at risk

|  | 0 | 1 year | 2 years | 3 years | 4 years | 5 years |
| --- | --- | --- | --- | --- | --- | --- |
| Risk score 0 | 23 | 17 | 8 | 5 | 4 | 3 |
| Risk score 1-2 | 25 | 8 | 3 | 0 | 0 | 0 |

When assessing outcomes using only age ≥ 75 years and CONUT score ≥ 4, which can be easily assessed preoperatively, the 1- and 3-year overall survival rates after recurrence to death were 73.0 % and 30.7 % (median survival time from recurrence to death: 1.4 years) in patients with risk score zero and 31.1 % and not available (median survival time from recurrence to death: 0.5 years) in those with risk score 1-2, respectively. The median time from recurrence to death of patients with risk score 1-2 was significantly shorter than patients risk score zero (P = 0.0010).
